# Supplementary material for: Extra Virgin Olive Oil Reduces Gut Permeability and Metabolic Endotoxemia in Diabetic Patients
Source: Nutrients. 2022 May 21;14(10):2153. doi: 10.3390/nu14102153 (PMC9145083; doi:10.3390/nu14102153)
Supplement: Supplementary file 1 [file nutrients-14-02153-s001.zip › nutrients-1716971-supplementary.pdf]

Supplementary Table S1. Vitamin E and total polyphenols content in EVOO**.**

**Compound**	**EVOO**
Vitamin E (mg/Kg)	75
Total polyphenols (mg/L GAE)	435
Saturated Fatty Acid (% m/m)	18.67
Mono-saturated Fatty Acid (% m/m)	66.6
Poli-saturated Fatty Acid (% m/m)	13.6
Peroxide (meq O2/kg)	<10
Oleuropein (mg/Kg)	4.0
Acidity (%)	0.34
Hydroxytyrosol (mg/g)	2.54
Tyrosol (mg/g)	7.89

Supplementary Table S2. Meal composition.

	**Proteins (g)**	**Lipids (g)**	**Carbohydrates (g)**	**Total fiber (g)**
Pasta (100g)	10.8	0.3	82.8	0
Chicken breast (150g)	35.4	2.1	0	0
Salad (80g)	1.44	0.32	1.76	1.5
Bread (80g)	6.56	0.4	54	0
Apple (200g)	0.4	0,6	22	3
Total g.	54.6	3.72	160.56	4.5
Kcal	218.4	33.48	642.24	9
